# Prevalence and characteristics of the Brugada electrocardiogram pattern in patients with arrhythmogenic right ventricular cardiomyopathy

**DOI:** 10.1002/joa3.12628

**Published:** 2021-08-30

**Authors:** Nobuhiko Ueda, Satoshi Nagase, Naoya Kataoka, Kenzaburo Nakajima, Tsukasa Kamakura, Mitsuru Wada, Kenichiro Yamagata, Kohei Ishibashi, Yuko Inoue, Koji Miyamoto, Takashi Noda, Takeshi Aiba, Chisato Izumi, Teruo Noguchi, Seiko Ohno, Kengo Kusano

**Affiliations:** ^1^ Division of Arrhythmia and Electrophysiology, Department of Cardiovascular Medicine National Cerebral and Cardiovascular Center Suita Japan; ^2^ Department of Advanced Arrhythmia and Translational Medical Science National Cerebral and Cardiovascular Center Suita Japan; ^3^ Second Department of Internal Medicine University of Toyama Toyama Japan; ^4^ Department of Bioscience and Genetics National Cerebral and Cardiovascular Center Suita Japan

**Keywords:** arrhythmogenic right ventricular cardiomyopathy, Brugada syndrome, cardiac death, depolarization abnormality, heart failure

## Abstract

**Background:**

Despite distinct pathophysiology, arrhythmogenic right ventricular cardiomyopathy (ARVC) and Brugada syndrome (BrS) exhibit overlapping phenotypes. We investigated the prevalence and characteristics of the Brugada electrocardiogram (ECG) pattern in ARVC patients.

**Methods:**

A total of 114 ARVC patients fulfilling the revised Task Force Criteria were enrolled. The Brugada ECG pattern was evaluated according to the consensus report on right precordial leads, and 1141 ECGs (median, 1; interquartile range, 1‐16 ECGs/patient) were analyzed.

**Results:**

Five patients (4%) showed a Brugada ECG pattern, which disappeared in four patients with ECGs recorded more than 2 years afterward. ARVC patients with the Brugada ECG pattern had a longer PQ interval (220 ± 62 ms vs 180 ± 35 ms, *P* = .02) and longer QRS duration (138 ± 25 ms vs 102 ± 23 ms, *P* < .001) than patients without the pattern. During follow‐up (median, 11.4; interquartile range, 5.5‐17.1 years), 19 ARVC patients experienced cardiac death and 29 experienced heart failure (HF) hospitalization. Kaplan‐Meier analysis determined that the Brugada ECG pattern increased the risk of cardiac death and HF hospitalization (log‐rank; *P* < .001, *P* < .001 respectively). The mean J‐point and S‐wave amplitudes of the Brugada ECG pattern were 0.29 ± 0.05 mV and 0.34 ± 0.21 mV, respectively, which were significantly lower than those of 26 age‐matched BrS patients with a previous ventricular fibrillation episode (0.66 ± 0.33 mV, *P* < .001 and 0.67 ± 0.39 mV, *P* = .02 respectively).

**Conclusion:**

The Brugada ECG pattern was infrequently encountered, was transient in ARVC patients, and was associated with a longer PQ interval, longer QRS duration, and cardiac events.

## Graphical abstract

Despite distinct pathophysiology, several reports have suggested overlapping phenotypes between arrhythmogenic right ventricular cardiomyopathy (ARVC) and Brugada syndrome. A Brugada ECG pattern was transiently recorded at least once in 5 out of 114 ARVC patients (4%). A transient Brugada ECG pattern (*) was associated with cardiac death in patients with ARVC.

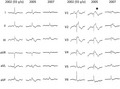

## 1 INTRODUCTION

Arrhythmogenic right ventricular cardiomyopathy (ARVC) is a myocardial disorder characterized by the morphological features of increased dimensions and wall motion abnormalities in the right ventricle (RV), fibrofatty replacement of the myocardium predominantly from RV subepicardium, and fatal ventricular arrhythmia (VA).^1^ Definite diagnostic criteria were established in the revised diagnostic Task Force Criteria (rTFC) published in 2010.^2^

Brugada syndrome (BrS) is characterized by a right precordial J‐ST segment elevation on electrocardiogram (ECG) and sudden cardiac death (SCD) from ventricular fibrillation (VF) without overt structural abnormality.^3^ Although the pathophysiology of BrS remains unclear, several hypotheses have been proposed. Initially, it was thought that ST elevation and VF appeared because of the abnormal dispersion of repolarization predominantly in the subepicardium. Recent studies have reported the fragmented and delayed potential in the right ventricular outflow tract (RVOT) epicardium and radiofrequency ablation for these potentials can suppress VF occurrence and cause the type 1 Brugada ECG to disappear.^4^, ^5^ Several imaging studies have also identified structural abnormalities.^6^, ^7^ The ECG diagnosis was established in the 2005 consensus report.^8^

Although ARVC and BrS are thought to be different diseases, as validated by the diagnostic criteria of each disease, some reports have suggested that there are overlapping characteristics between the two.^9^, ^10^, ^11^, ^12^, ^13^, ^14^ Epicardial abnormality is the primary pathophysiological feature in both ARVC and BrS. Similar features, especially those pertaining to the ECG, can be provoked in some cases. The manifestation of a Brugada phenotype in many ARVC patients with long‐term follow‐up has not yet been reported. Therefore, the purpose of this study was to investigate the prevalence and clinical significance of the Brugada ECG pattern in patients with ARVC.

## 2 METHODS

### 2.1 Study group

In this single‐center cohort study, we evaluated 114 consecutive ARVC patients who fulfilled the rTFC. In accordance with the rTFC, 97 ARVC patients (85%) were categorized as definite, and 17 patients (15%) were categorized as borderline. The median follow‐up duration was 11.4 (interquartile range, 5.5‐17.1) years. A total of 1141 ECGs (median, 1; interquartile range, 1‐16 ECGs/ARVC patient) were analyzed. In 37 ARVC patients, right precordial leads on the upper one and two intercostal spaces were additionally recorded. Furthermore, 26 age‐matched BrS patients (mean age: 52.6 ± 14.7 years) with a spontaneous type 1 ECG and VF episode were evaluated for a comparison of the amplitude of the Brugada‐type ECG compared to that of ARVC patients. This study was approved by the Institutional Review Board (IRB) of the National Cerebral and Cardiovascular Center, Suita, Japan (R19114). The IRB approved this study and waived informed consent by disclosing information about the study to the public and ensuring that study subjects had an opportunity to refuse to participate.

### 2.2 Definition of Brugada ECG pattern

A positive Brugada ECG pattern was defined as a type 1 or type 2 ECG with a J‐point elevation ≥2 mm in lead V1 to V3, in accordance with previous reports.^15^, ^16^ The J‐point was defined as the junction between the end of the QRS complex and the beginning of the ST segment determined in lead V5 or V6. A type 1 ECG was defined as a J‐point elevation ≥2 mm, followed by a negative T wave. A type 2 ECG was defined as a J‐point elevation ≥2 mm, with a gradual ST‐segment descent ≥1 mm, followed by a positive or biphasic T wave. A type 2 ECG was also defined as a β angle ≥58° and a duration of the base of the triangle of r' at 5 mm from the high take‐off ≥3.5 mm.^17^ Analysis was performed in a blinded manner by two independent cardiologists (SN and NU).

### 2.3 Clinical data

Clinical data were collected from all patients and included their age, gender, echocardiographic data, such as the left ventricular ejection fraction (LVEF), right ventricular ejection fraction (RVEF) measured by magnetic resonance imaging (MRI), morphologies of ECG such as T‐wave inversion in the right precordial leads and inferior leads, epsilon wave, complete right bundle branch block, J wave, and parameters of an ECG, such as QRS duration and PQ interval. Because a class III antiarrhythmic drug was administered in approximately 40% of patients, QT, corrected QT and Tpeak‐Tend intervals were not measured. An epsilon wave was defined as reproducible low‐amplitude signals between the ends of the QRS complex and the onset of the T wave in the right precordial leads.^2^ Terminal activation duration was measured from the nadir of the S wave to the end of all depolarization deflections. An inferolateral J wave was defined as a J‐point elevation of ≥0.1 mV above the baseline that was either notched (a positive J deflection at the QRS‐complex/ST segment transition) or slurred (a smooth transition from QRS to the ST segment) in at least two consecutive leads.^18^ Fragmented QRS was defined as deflections at the beginning of the QRS complex, on top of the R wave, or in the nadir of the S wave in at least one lead.^19^ The measurement of the ECG parameters was performed at the time of the first ECG recording. Clinical outcomes included the incidence of cardiac death, VAs, and hospitalization because of heart failure (HF). VA was defined as sustained ventricular tachycardia (VT), VF, or necessary intervention with an implantable cardioverter‐defibrillator during follow‐up. Hospitalization for HF was defined as the sudden or gradual onset of the signs or symptoms of New York Heart Association class 3 or 4 HF, which required an unplanned hospitalization without subsequent VT/VF episodes. SCD was defined as any natural death that occurred instantaneously or within 1 hour after symptom onset. Cardiac death included SCD, HF‐related death, and heart transplantation.

An analysis of genetic variants for ARVC, including the desmosomal genes plakophilin‐2 (*PKP2*), desmoplakin (*DSP*), desmocollin‐2 (*DSC2*), desmoglein‐2 (*DSG2*), and plakoglobin (*JUP*), was performed in 18 patients, as reported previously (Document S1).^20^

### 2.4 Follow‐up

Patients were followed up in our hospital for a median duration of 11.4 (interquartile range, 5.5‐17.1) years. Follow‐up information was obtained from an implantable cardioverter‐defibrillator and pacemaker follow‐up charts, hospital records, and outpatient evaluations.

### 2.5 Statistical analyses

The results are summarized as the mean ± SD if normally distributed or as the median (interquartile range) if not normally distributed. The categorical data were expressed as counts and percentages. Categorical differences between groups were evaluated using a χ2 test or Fisher's exact test as appropriate. Continuous variables were compared using the Wilcoxon rank‐sum test or the Kruskal‐Wallis test. A univariate Cox regression analysis was performed to evaluate significant predictors of events. Survival curves were determined by the Kaplan‐Meier method and analyzed by the log‐rank test. A value of *P* < .05 was taken as the threshold for statistical significance. All analyses were performed using JMP 12 software (SAS Institute, Cary, NC, USA).

## 3 RESULTS

### 3.1 Clinical characteristics

**FIGURE 1:**
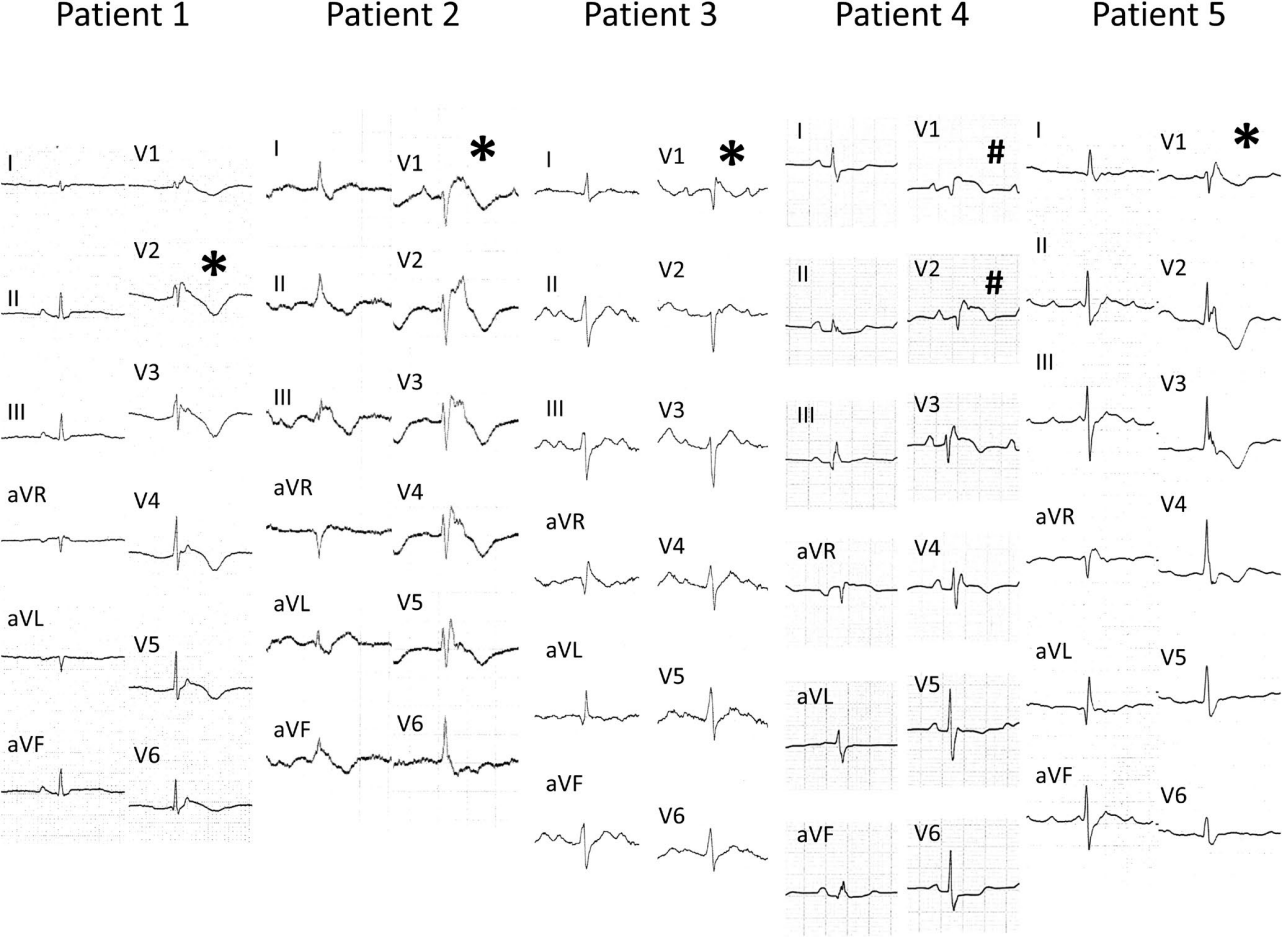
Brugada ECG pattern in all five patients. *, type 1 ECG; #, type 2 ECG

**FIGURE 2:**
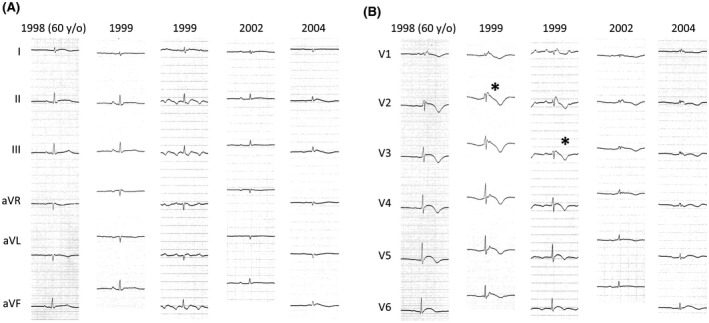
This male patient (Patient 1) was diagnosed with ARVC in 1998, at the age of 60 years old. He was repeatedly hospitalized for heart failure. His left ventricular ejection fraction was 54%, and his right ventricular ejection fraction was 19%. In 2004, sustained ventricular tachycardia appeared, and he died of heart failure. In 1999, a type 1 ECG was observed (*). After 1999, the type 1 ECG disappeared as the QRS amplitude decreased

**FIGURE 3:**
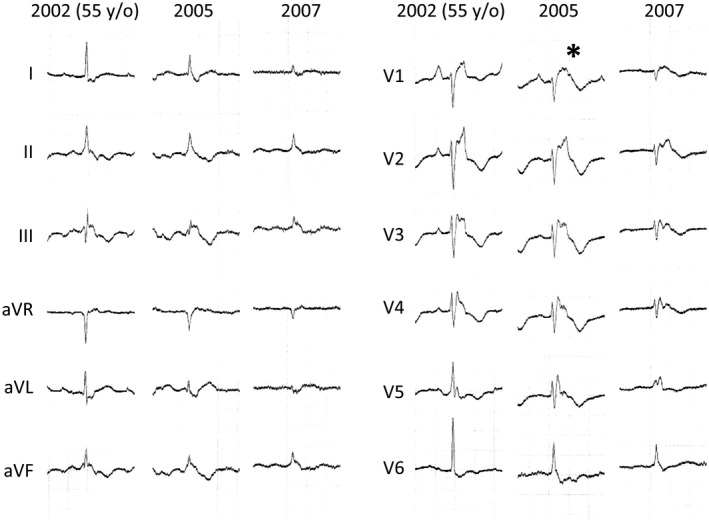
This female patient (Patient 2) was diagnosed with ARVC in 2002, at the age of 55 years old. She was repeatedly hospitalized for heart failure. Her left ventricular ejection fraction was 50%, and her right ventricular ejection fraction was 19%. In 2007, she died of heart failure. In 2005, a type 1 ECG was observed (*) as the right bundle branch block was exacerbated. In 2007, the type 1 ECG became ambiguous as the QRS amplitude decreased

A Brugada ECG pattern was transiently recorded at least once in 5 of 114 patients (4%). Figures 1, 2, 3 and Figures S1‐S3 are representative ECGs. Among them, a type 1 ECG was recorded in four patients, and a type 2 ECG was recorded in one patient. After its appearance, the Brugada ECG pattern disappeared in all four patients in whom an ECG was recorded more than 2 years after the initial recording of the pattern. Baseline characteristics among ARVC patients with and without a Brugada ECG pattern are presented in Table 1. The PQ interval (220 ± 62 ms vs 180 ± 35 ms, *P* = .02) and QRS duration (138 ± 25 ms vs 102 ± 23 ms, *P* < .001) in patients with a Brugada ECG pattern were significantly longer than in patients without a Brugada ECG pattern. More information about the 5 patients with a Brugada ECG pattern is provided in Documents S2 and S3. A genetic variant associated with ARVC was found in 15 of 18 patients (5 with *PKP2* and 10 with *DSG2*, Table S1); however, genetic analysis was not performed in any of the 5 patients with a Brugada ECG pattern.

**TABLE 1:** Comparison of characteristics between arrhythmogenic right ventricular cardiomyopathy patients with and without the Brugada pattern on electrocardiogram

Clinical characteristics	All	Brugada pattern (+)	Brugada pattern (−)	*P* value
Number, n (%)	114 (100)	5 (4)	109 (96)	
Male, n (%)	85 (75)	3 (60)	82 (75)	.47
Age at enrolment (years)	46.5 (33.8‐56.3)	55.0 (48.5‐61.5)	46.0 (33.0‐56.0)	.14
BSA (m^2^)	1.7 (1.5‐1.8)	1.7 (1.5‐1.8)	1.7 (1.6‐1.8)	.74
Diagnosis based on rTFC				.29
Definite, n (%)	102 (89)	5 (100)	97 (89)	
Borderline, n (%)	12 (11)	0 (0)	12 (11)	
Cardiac function				
LVEF (%)	55 (44.8‐62.3)	55.0 (42.5‐63.5)	55.0 (44.5‐62.0)	.84
RVEF (%)	30.7 ± 10.6	24.0 ± 12.4	31.1 ± 10.6	.15
RV asynergy/aneurysm by RV angiography/MRI, n (%)	58/102 (57)	5/5 (100)	53/97 (55)	.016
ECG				
PQ interval (ms)	182 ± 37	220 ± 62	180 ± 35	.020
QRS duration (ms)	104 ± 24	138 ± 25	102 ± 23	.0008
CRBBB, n (%)	22 (19)	3 (60)	19 (17)	.039
Fragmented QRS, n (%)	52 (46)	3 (60)	49 (45)	.51
T‐wave inversion in leads V1‐V3, n (%)	87 (76)	3 (60)	84 (77)	.41
T‐wave inversion in leads II/III/aVF, n (%)	50 (44)	1 (20)	49 (45)	.25
Prolonged TAD, n (%)	72/92 (78)	2/2 (100)	70/90 (78)	.32
ε wave, n (%)	23 (20)	2 (40)	21 (19)	.30
J wave, n (%)	17 (15)	1 (20)	16 (15)	.75
Positive in SAECG, n (%)	102/111 (92)	4/4 (100)	98/107 (92)	.41
EPS inducibility, n (%)	60/91 (66)	3/3 (100)	57/88 (65)	.11
Fibrofatty replacement of myocardium on EMB, n (%)	52/82 (63)	3/4 (75)	49/78 (63)	.61
History of catheter ablation, n (%)	10 (9)	0 (0)	10 (9)	.29
Catheter ablation during follow‐up, n (%)	54 (47)	1 (17)	53 (49)	.33
ICD implantation during follow‐up, n (%)	42 (37)	1 (20)	41 (38)	.40
Medication during follow‐up				
Amiodarone, n (%)	36 (32)	2 (40)	34 (31)	.68
Sotalol, n (%)	21 (18)	2 (40)	19 (17)	.25
β‐blockers, n (%)	60 (53)	0 (0)	60 (55)	.0054
Family history of ARVC, n (%)	3 (3)	0 (0)	3 (3)	.60

Values are presented as mean ± SD, median (interquartile range), or n (%).

ARVC, arrhythmogenic right ventricular cardiomyopathy; BSA, body surface area; CRBBB, complete right bundle branch block; ECG, electrocardiogram; EMB, endomyocardial biopsy; EPS, electrophysiological study; ICD, implantable cardioverter‐defibrillator; LVEF, left ventricular ejection fraction; MRI, magnetic resonance imaging; rTFC, revised Task Force Criteria; RV, right ventricular; RVEF, right ventricular ejection fraction; SAECG, signal‐averaged electrocardiogram; TAD, terminal activation duration.

### 3.2 Clinical outcomes

**FIGURE 4:**
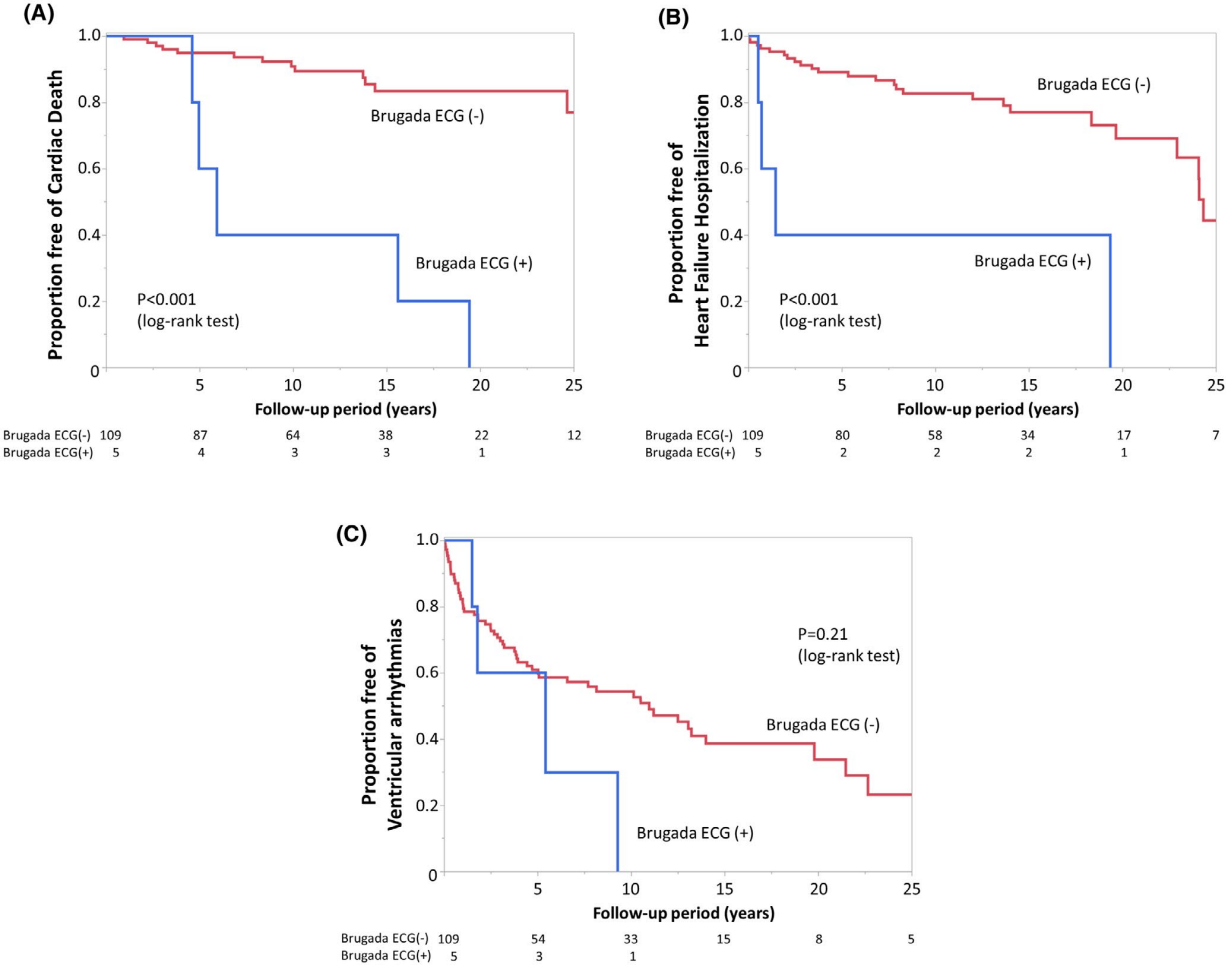
Kaplan‐Meier analysis of freedom from cardiac death (A), heart failure (HF) hospitalization (B), and fatal ventricular arrhythmia (VA, C) during the follow‐up period in patients with and without a Brugada ECG pattern. Patients with a Brugada pattern ECG (blue line) had a significantly higher risk of cardiac death (log‐rank, *P* < .001) and HF hospitalization (log‐rank, *P* < .001) compared to patients without a Brugada ECG pattern (red line). The occurrence of VA was similar between the two groups (log‐rank, *P* = .21)

During the follow‐up period, 19 patients (5 with and 14 without a Brugada ECG pattern) died because of cardiac causes, 29 patients (4 with and 25 without a Brugada ECG pattern) experienced HF hospitalization, and 61 patients (4 with and 57 without a Brugada ECG pattern) had VAs. Kaplan‐Meier analysis revealed that patients with a Brugada ECG pattern were at a significantly higher risk for cardiac death (log‐rank, *P* < .001; Figure 4A) and HF hospitalization (log‐rank, *P* < .001; Figure 4B) than patients without a Brugada ECG pattern. The occurrence of VA was similar between the two groups (log‐rank, *P* = .21; Figure 4C).

### 3.3 Univariate analysis

As presented in Table 2, univariate analysis revealed that a positive Brugada ECG pattern was associated with cardiac death (hazard ratio [HR]: 9.46, 95% confidence interval [CI]: 3.31‐27.0, *P* < .001) and HF hospitalization (HR: 6.97, 95% CI: 2.35‐20.7, *P* = .005).

**TABLE 2:** Univariate analyses for prediction of: (A) cardiac death during follow‐up and (B) heart failure hospitalization during follow‐up

(A) Cardiac death during follow‐up	Univariate analysis		
	HR	95% CI	*P* value
Gender (male)	0.44	0.17‐1.15	.11
Age at enrolment	1.03	0.99‐1.07	.17
BSA*100 (1 m^2^ increase)	0.96	0.93‐0.99	.008
Cardiac function			
LVEF (1% increase)	0.96	0.93‐0.99	.005
RVEF (1% increase)	0.90	0.84‐0.96	.0002
RV asynergy/aneurysm by RV angiography/MRI	0.98	0.37‐2.55	.96
ECG			
Brugada ECG pattern	9.46	3.31‐27.0	<.0001
PQ interval (1‐ms increase)	1.01	1.01‐1.02	.22
QRS duration (1‐ms increase)	1.02	1.00‐1.04	.019
CRBBB	1.77	0.70‐4.52	.24
Fragmented QRS	1.36	0.55‐3.35	.51
T‐wave inversion in leads V1‐V3	0.74	0.28‐1.96	.55
T‐wave inversion in leads II/III/aVF	0.72	0.27‐1.91	.51
Prolonged TAD	0.97	0.21‐4.44	.97
ε wave	1.52	0.58‐3.99	.40
J wave	0.38	0.051‐2.86	.28
EPS inducibility	0.63	0.18‐2.28	.49
History of catheter ablation	0.46	0.061‐3.47	.40

(B) Heart failure hospitalization during follow‐up	Univariate analysis		
	HR	95%CI	*P* value
Gender (male)	0.57	0.26‐1.26	.18
Age at enrolment	1.03	1.00‐1.06	.05
BSA*100 (1 m^2^ increase)	0.98	0.96‐1.01	.20
Cardiac function			
LVEF (1% increase)	0.96	0.94‐0.98	.0006
RVEF (1% increase)	0.93	0.89‐0.97	.0008
RV asynergy/aneurysm by RV angiography/MRI	1.09	0.49‐2.40	.84
ECG			
Brugada ECG pattern	6.97	2.35‐20.7	.005
PQ interval (1‐ms increase)	1.01	1.01‐1.02	.001
QRS duration (1‐ms increase)	1.01	1.00‐1.02	.17
CRBBB	1.35	0.60‐3.07	.48
Fragmented QRS	0.78	0.37‐1.63	.50
T‐wave inversion in leads V1‐V3	0.90	0.39‐2.03	.79
T‐wave inversion in leads II/III/aVF	0.73	0.34‐1.58	.42
Prolonged TAD	0.79	0.26‐2.40	.69
ε wave	1.87	0.88‐3.97	.12
J wave	0.39	0.09‐1.63	.14
EPS inducibility	0.68	0.26‐1.74	.43
History of catheter ablation	1.60	0.60‐4.23	.37

### 3.4 Comparison between ARVC and age‐matched BrS

The comparison between ARVC patients with a Brugada ECG pattern and age‐matched BrS patients is presented in Table 3. The amplitudes of the J‐point and S wave showing a Brugada ECG pattern were significantly lower in ARVC patients compared to BrS patients (0.29 ± 0.05 mV vs 0.66 ± 0.33 mV, *P* < .001 and 0.34 ± 0.21 mV vs 0.67 ± 0.39 mV, *P* = .017 respectively).

**TABLE 3:** Comparison of characteristics between arrhythmogenic right ventricular cardiomyopathy patients with the Brugada pattern on electrocardiogram and Brugada syndrome patients

Clinical characteristics	ARVC	Brugada	*P* value
Age at enrolment (years)	55.0 ± 7.3	52.6 ± 14.7	.73
ECG			
PQ interval (ms)	210 (170‐278)	180 (163‐208)	.13
QRS duration (ms)	138 ± 25	118 ± 31	.18
Complete right bundle brunch block, n (%)	3 (60)	8 (31)	.22
Fragmented QRS, n (%)	3 (60)	8 (31)	.22
T‐wave inversion in leads V1‐V3, n (%)	3 (60)	16 (62)	.95
T‐wave inversion in leads II/III/aVF, n (%)	1 (20)	3 (12)	.62
J wave in inferior/lateral lead, n (%)	1 (20)	4 (15)	.80
Positive in SAECG, n (%)	4/4 (100)	8/13 (62)	.07
J‐point amplitude of Brugada pattern (mV)	0.29 ± 0.05	0.66 ± 0.33	<.001
S‐wave amplitude of Brugada pattern (mV)	0.34 ± 0.21	0.67 ± 0.39	.017
Episode of VF, n (%)	0 (0)	26 (100)	<.001
Episode of monomorphic VT, n (%)	4 (80)	0 (0)	<.001

Values are presented as mean ± SD, median (interquartile range), or n (%).

VF, ventricular fibrillation; VT, ventricular tachycardia.

## 4 DISCUSSION

### 4.1 Main findings

To the best of our knowledge, this is the first study to investigate the prevalence and characteristics of the Brugada ECG pattern in patients with ARVC. This study found that the prevalence of the Brugada ECG pattern was quite low despite a long‐term follow‐up period and repeated ECG with upper intercostal recordings. The appearance of the Brugada ECG pattern was transient. The Brugada ECG pattern in ARVC patients was associated with a longer PQ interval, longer QRS duration, and cardiac events, including cardiac death and HF hospitalization.

### 4.2 Overlap between ARVC and BrS

Although ARVC and BrS were diagnosed based on different criteria, some studies have shown that overlapping features are observed in some patients. Martini et al. first described three cases resuscitated from VF with right precordial ST‐T segment elevation and right ventricular abnormalities.^21^ Structural right ventricular abnormalities and conduction defects concomitant with ARVC were discovered in a series of autopsies of patients with ST‐segment elevation in the right precordial leads and an episode of SCD.^9^, ^10^ Corrado et al. reported that right precordial ST‐segment elevation was found in 13 (14%) of 273 young SCD victims on their last recorded ECG.^10^ These data indicate that structural right ventricular abnormalities can be associated with right precordial ST‐segment elevation in patients with ARVC.

### 4.3 Mechanism of Brugada ECG pattern in ARVC

Two hypotheses for the pathophysiological mechanism of BrS have been reported, the repolarization hypothesis^22^ and the depolarization hypothesis.^23^, ^24^ The repolarization hypothesis suggests that a functional change causes a subepicardial abbreviation of an action potential and a transmural dispersion of repolarization, which causes an ST‐segment elevation.^22^ However, the depolarization hypothesis suggests that a structural change in the subepicardium causes conduction abnormalities based on interstitial fibrosis and results in ECG changes^23^ that lead to an activation delay or excitation failure by current‐to‐load mismatch, which underlies BrS.^25^ Although the mechanism of the overlapping features in ARVC patients and the Brugada ECG pattern remains unclear, both repolarization and depolarization abnormalities can be serious.

In this study, the QRS duration in patients with a Brugada ECG pattern was longer than that in patients without a Brugada ECG pattern. A Brugada ECG pattern appeared transiently from early to mid‐term. Further disease progression may have led to a disappearance of the Brugada ECG pattern as the conduction delay worsened. Cerrone et al. reported that plakophilin‐2 mutations led to disruption of the desmosome, which caused a reduction in sodium channels and a reduction in sodium current.^11^, ^12^ This causes an outward shift in the balance of the epicardial action potential current in the RVOT, which is likely the underlying mechanism for the manifestation of the Brugada ECG pattern in the early to mid‐phase of ARVC. With advancing ARVC, the epicardial cells undergo apoptosis, which can lead to a decrease in the BrS phenotype. Similarly, in BrS, Nademanee et al reported that a transient Brugada ECG pattern may initially indicate epicardial interstitial fibrosis and reduced gap junction expression.^26^ As the disease progresses, fibrosis and gap junction disturbance may develop transmurally, leading to a lower transmural voltage gradient. This may result in the disappearance of the BrS ECG pattern. Disease progression is likely associated with a pronounced low voltage area in the RVOT, leading to the development of low QRS voltage on surface ECG and the disappearance of the Brugada ECG pattern.

### 4.4 Relationship between the Brugada ECG pattern and clinical events

This study showed that the Brugada ECG pattern was associated with a longer PQ interval, longer QRS duration, and cardiac events, including cardiac death and HF hospitalization, in ARVC patients. Right ventricular asynergy or aneurysm detected by angiography or MRI was significantly frequent in patients with the Brugada ECG pattern (Table 1). The RVEF tended to be lower in patients with the Brugada ECG pattern. Severe dysfunction of the RV is likely associated with the Brugada ECG pattern and clinical adverse event.

### 4.5 Comparison of ECG between ARVC and BrS patients

This study found that the amplitudes of the J‐ST level and the S wave of the Brugada ECG pattern in ARVC patients were lower than those in BrS patients with VF episodes. We speculate that because a depolarization abnormality is more prominent in ARVC and is limited in BrS, the amplitudes of the J‐ST level and S wave were smaller in ARVC patients. However, it is possible that the mechanisms underlying the development of the Brugada ECG pattern in ARVC and BrS are not identical. The morphology of the Brugada ECG pattern in ARVC was somewhat different and atypical compared to the typical type 1 or type 2 ECG in patients with BrS. Fatal arrhythmia was VT dominant in ARVC patients but was VF dominant in BrS patients (Table 3).^27^ The impact on the changes in autonomic tone or heart rate will be valuable in identifying the differences in ECG characteristics between ARVC and BrS. Further investigation is necessary to understand the mechanism of characteristic J‐ST elevation in ARVC and BrS.

The significance of ECG in the diagnosis and risk assessment of ARVC was reported by Neto and Luna.^28^ They also mentioned that specific ECG findings in ARVC patients should be carefully evaluated because of high interobserver variability and disagreement between experts. Thus, extreme care should be taken in ECG analysis in patients with ARVC.

### 4.6 Limitations

This was a single‐center, retrospective study. The relatively small number of ARVC patients may therefore limit the interpretation of these results. Moreover, because of the low incidence of a positive Brugada‐type ECG, we should be cautious with statistical interpretations and clinical utility. A sodium‐channel blocker test was not performed to unmask the Brugada ECG pattern because of safety concerns. The additional recording of right precordial leads on the upper intercostal area was conducted in a small number of patients. Because daily and circadian fluctuation of J‐ST morphology is common in BrS and the Brugada ECG appeared transiently, there is a possibility that the detection rate was underestimated. The number of patients who underwent genetic testing for ARVC was low, and no ARVC patients with a Brugada ECG pattern underwent genetic analysis. Some ECGs with the Brugada pattern in ARVC patients showed an unrepresentative configuration for BrS patients. The mechanism of the Brugada ECG pattern was not evaluated in this study and the association with repolarization abnormality was unclear. Further prospective, multicenter studies, which include a larger number of ARVC patients, are warranted to confirm these findings.

## 5 CONCLUSIONS

In ARVC patients, the Brugada ECG pattern was infrequently encountered. This pattern appeared transiently and disappeared during follow‐up. A Brugada ECG pattern increased the risk of cardiac death and HF hospitalization in patients with ARVC.

## 6 IRB INFORMATION

This study was approved by the IRB of the National Cerebral and Cardiovascular Center, Suita, Japan (R19114). The IRB approved this study and waived informed consent by disclosing information about the study to the public and ensuring that the patients had the opportunity to refuse to participate.

## Supporting information

Fig S1‐S3

Table S1

Supplementary Material

## Data Availability

De‐identified participant data will not be shared because the dataset contains sensitive and potentially identifying patient information.
