# Supplementary material for: Prevalence and characteristics of the Brugada electrocardiogram pattern in patients with arrhythmogenic right ventricular cardiomyopathy
Source: J Arrhythm. 2021 Aug 30;37(5):1173–83. doi: 10.1002/joa3.12628 (PMC8485808; doi:10.1002/joa3.12628)
Supplement: Supplementary file 1 — Fig S1‐S3 [file JOA3-37-1173-s002.docx]

**Supplementary Figure**

**Figure S1**

**
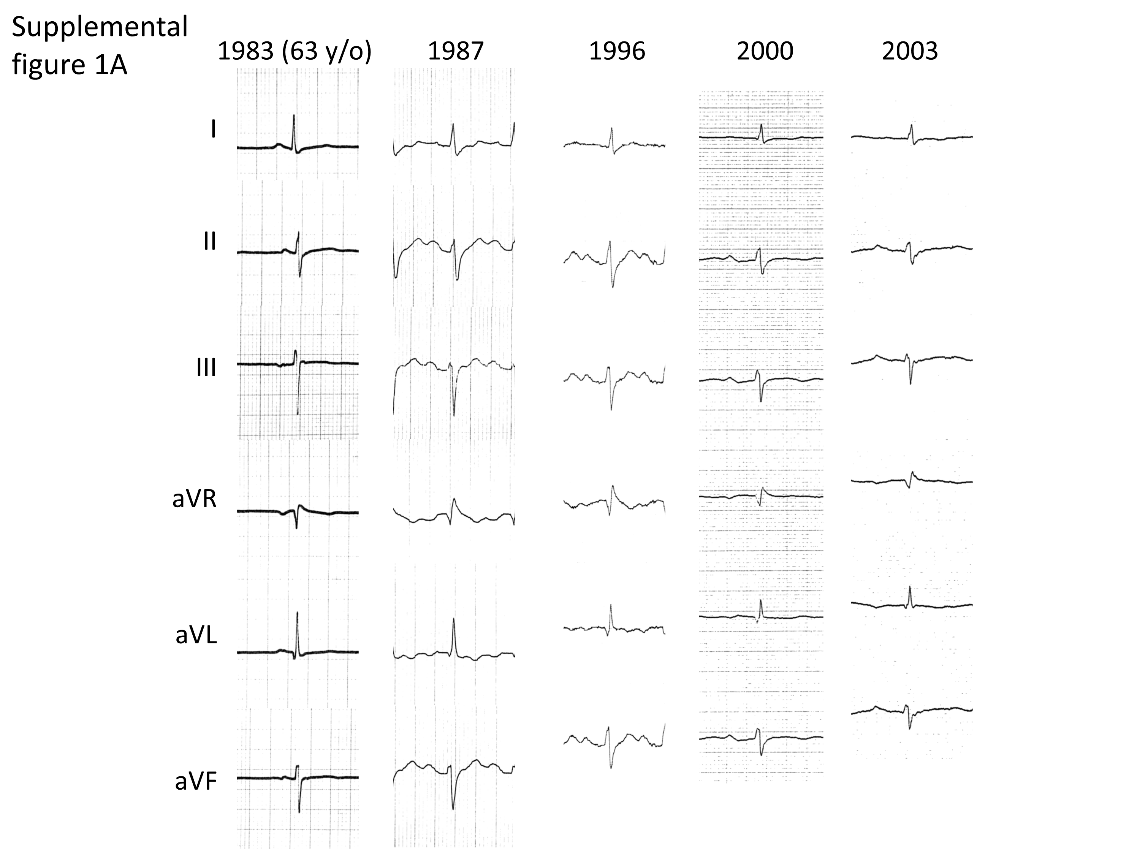

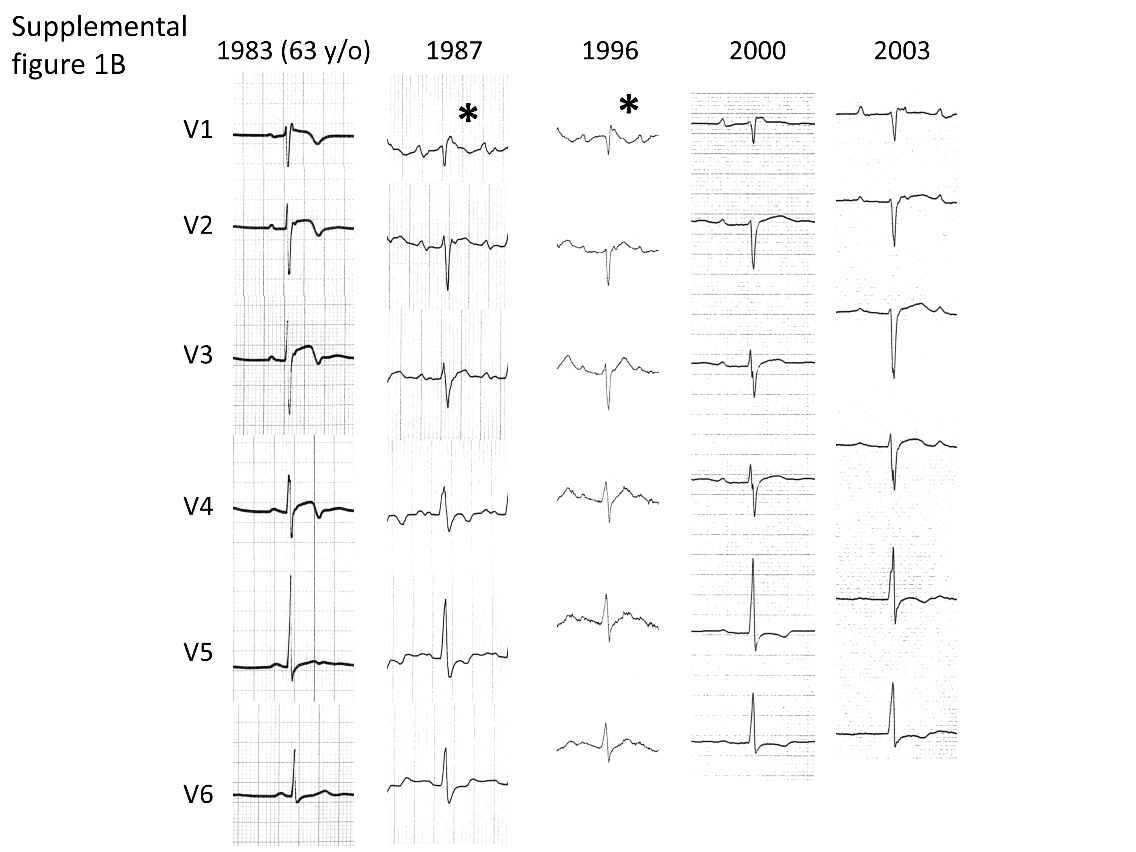
**

**Figure S1:**

This male patient (Patient 3) was diagnosed with ARVC in 1978 at the age of 58 years old. He was hospitalized repeatedly for heart failure. Sustained ventricular tachycardia was also observed. Left ventricular ejection fraction was 30%, and right ventricular ejection fraction was 45%. In 2003, he died suddenly. In 1987 and 1996, a type 1 ECG was observed (*).

**Figure S2**

**
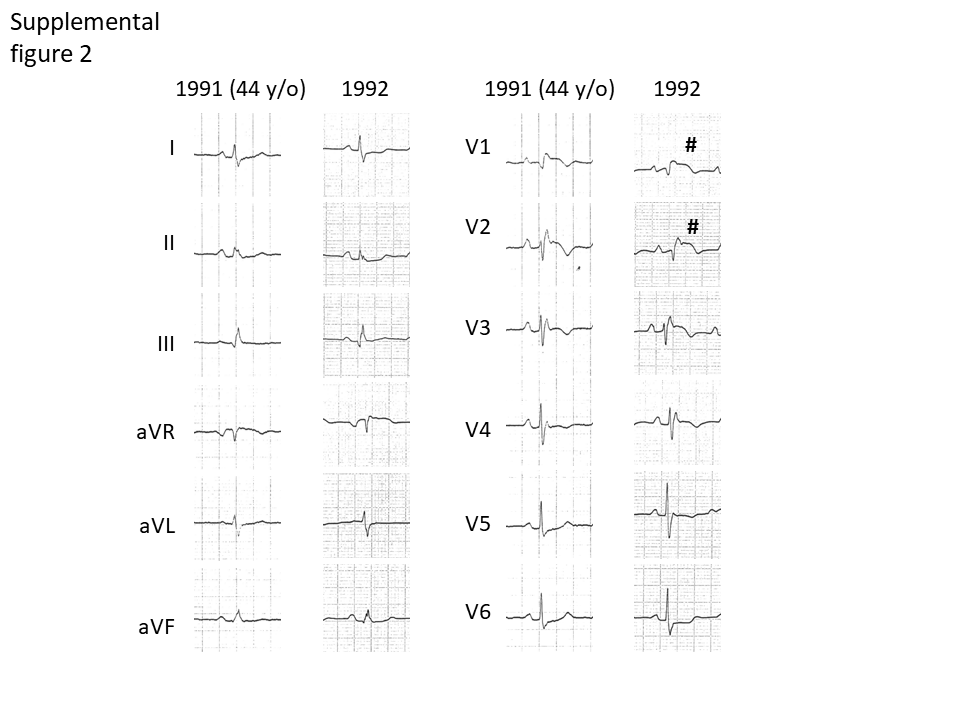
**

**Figure S2**

This male patient (Patient 4) was diagnosed with ARVC in 1991 at the age of 44 years old. Sustained ventricular tachycardia was observed. Left ventricular ejection fraction was 55%, and right ventricular ejection fraction was 15%. In 1995, he died of myocardial infarction and heart failure. In 1992, a type 2 ECG was observed (#).

**Figure S3**


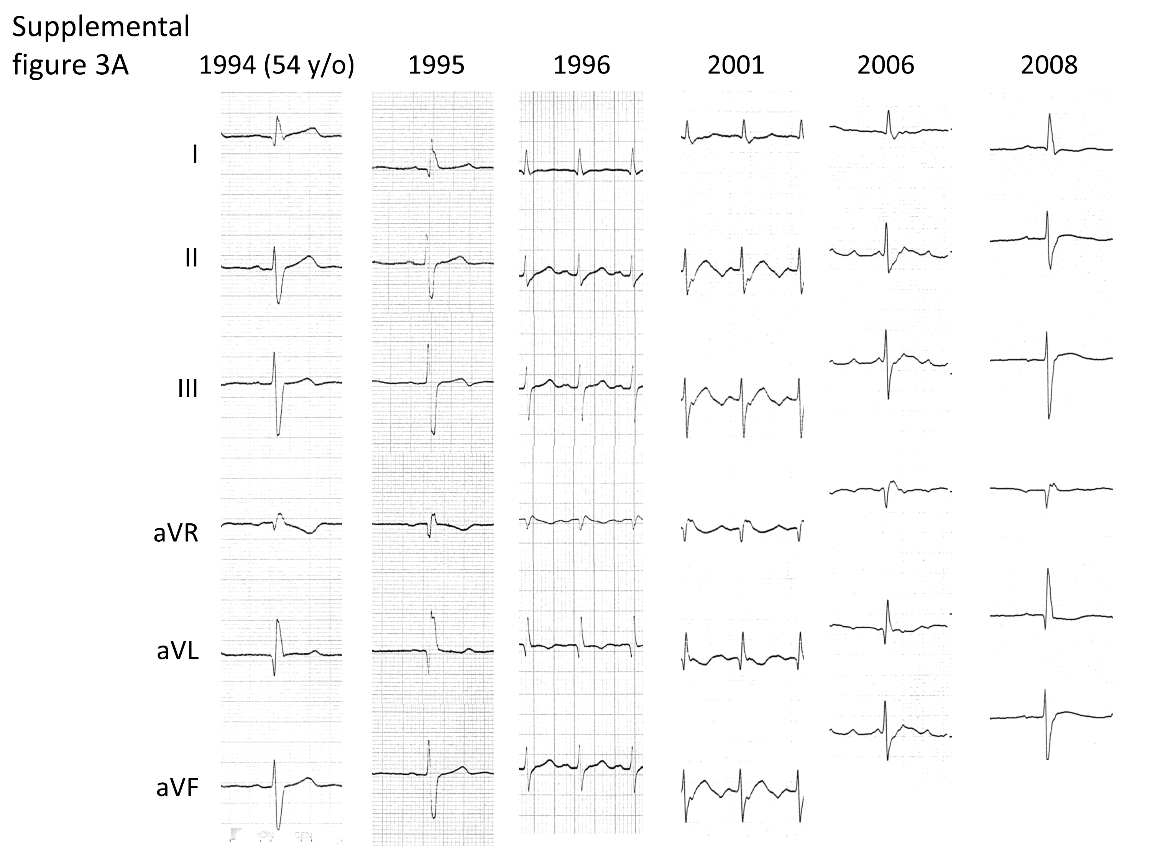


**
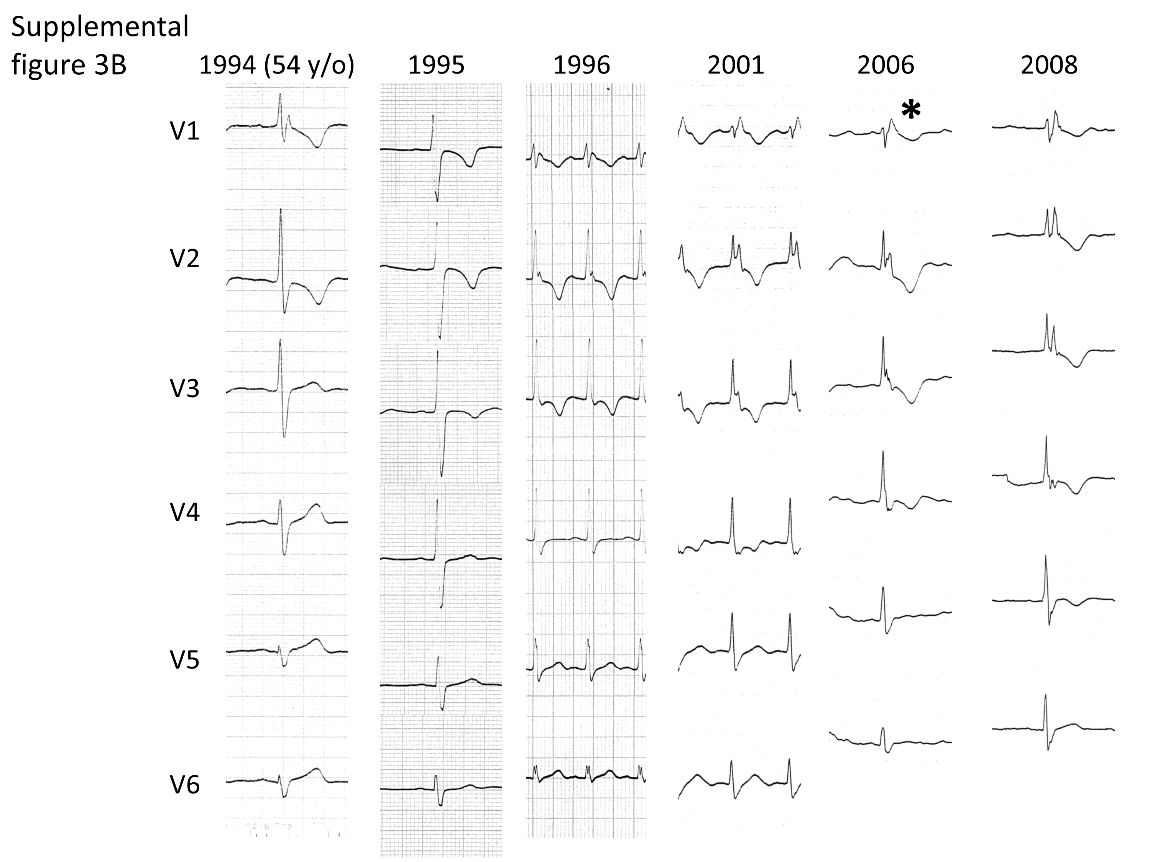
**

**Figure S3:**

This female patient (Patient 5) was diagnosed with ARVC in 1992 at the age of 52 years old. She was hospitalized repeatedly for heart failure. Sustained ventricular tachycardia was also observed. Left ventricular ejection fraction was 50%, and right ventricular ejection fraction was 25%. In 2009, she died of heart failure. In 2006, a type 1 ECG was observed (*).
