# Supplementary material for: Prevalence and characteristics of the Brugada electrocardiogram pattern in patients with arrhythmogenic right ventricular cardiomyopathy
Source: J Arrhythm. 2021 Aug 30;37(5):1173–83. doi: 10.1002/joa3.12628 (PMC8485808; doi:10.1002/joa3.12628)
Supplement: Supplementary file 2 — Table S1 [file JOA3-37-1173-s001.docx]

**Supplementary Table S1:**  Gene and clinical characteristics

| No. | Gender | Age at enrollment | Gene | Task force criteria | | | | | |  | Diagnosis | |
| --- | --- | --- | --- | --- | --- | --- | --- | --- | --- | --- | --- | --- |
|  |  |  |  | RV function | Tissue | Repolarization | Depolarization / Conduction | Arrhythmia | Family history |  | Major / minor | category |
| 1 | m | 38 | PKP2 |  | A | A |  | I |  |  | 2 / 1 | definite |
| 2 | m | 41 | PKP2 | A | A | A | A | I |  |  | 4 / 1 | definite |
| 3 | m | 26 | PKP2 | A |  |  |  | I |  |  | 1 / 1 | borderline |
| 4 | f | 22 | PKP2 | A | A | A |  | I |  |  | 3 / 1 | definite |
| 5 | f | 13 | PKP2 | A | I |  | I | I |  |  | 1 / 3 | definite |
| 6 | m | 40 | DSG2 | A |  |  | I | I |  |  | 1 / 2 | definite |
| 7 | m | 24 | DSG2 | A |  |  | A | I |  |  | 2 / 1 | definite |
| 8 | m | 52 | DSG2 | A | A |  | I | I |  |  | 2 / 2 | definite |
| 9 | f | 40 | DSG2 | I | I |  |  | I |  |  | 0 / 3 | borderline |
| 10 | m | 58 | DSG2 |  | I | A | A | I |  |  | 2 / 2 | definite |
| 11 | f | 19 | DSG2 | A | I |  | I |  |  |  | 1 / 2 | definite |
| 12 | m | 17 | DSG2 | A | A | A |  | A |  |  | 4 / 0 | definite |
| 13 | m | 44 | DSG2 | A | A |  |  |  |  |  | 2 / 0 | definite |
| 14 | m | 38 | DSG2 | A | A | A | I |  |  |  | 3 / 1 | definite |
| 15 | m | 16 | DSG2 | A | I | A |  |  |  |  | 2 / 1 | definite |

RV=right ventricle, A=major criteria with the revised diagnostic Task Force Criteria (rTFC), I=minor criteria with the rTFC.
