# Supplementary material for: Prevalence and characteristics of the Brugada electrocardiogram pattern in patients with arrhythmogenic right ventricular cardiomyopathy
Source: J Arrhythm. 2021 Aug 30;37(5):1173–83. doi: 10.1002/joa3.12628 (PMC8485808; doi:10.1002/joa3.12628)
Supplement: Supplementary file 3 — Supplementary Material [file JOA3-37-1173-s003.docx]

**Supplementary Doc. 1**

**Method of Genetic analysis:**

Genomic DNA was isolated from venous blood lymphocytes as described previously ^1^. Genotyping was performed according to a bidirectional direct deoxyribonucleic acid sequencing method targeting a comprehensive open reading frame/splice site mutational analysis of four major ARVD/C susceptibility genes ^2^ : PKP2, encoding plakophilin 2; DSP, encoding desmoplakin; DSG2, encoding desmoglein 2; and DSC2, encoding desmocollin 2. To screen for non-desmosomal genes, all probands were genotyped for LMNA and SCN5A, encoding lamin A/C and voltage gated sodium channel alpha subunit type V, respectively. The cDNA sequences of PKP2, DSP, DSG2, DSC2, LMNA, and SCN5A were based on the GenBank reference sequences NM_004572.3, NM_001008844.2, NM_001943.4, NM_004949.4, NM_001257374.2, and NM_00335.4, respectively.

***References***

1. Ohno S, Nagaoka I, Fukuyama M, Kimura H, Itoh H, Makiyama T, Shimizu A, Horie M. Age-dependent clinical and genetic characteristics in japanese patients with arrhythmogenic right ventricular cardiomyopathy/dysplasia. Circulation journal : official journal of the Japanese Circulation Society. 2013;77:1534-1542

2. Rigato I, Bauce B, Rampazzo A, Zorzi A, Pilichou K, Mazzotti E, Migliore F, Marra MP, Lorenzon A, De Bortoli M, Calore M, Nava A, Daliento L, Gregori D, Iliceto S, Thiene G, Basso C, Corrado D. Compound and digenic heterozygosity predicts lifetime arrhythmic outcome and sudden cardiac death in desmosomal gene-related arrhythmogenic right ventricular cardiomyopathy. Circulation. Cardiovascular genetics. 2013;6:533-542

**Supplementary Doc. 2**

**Ventricular tachyarrhythmia induced in EPS and clinically recognized:**

# Patient 1:

EPS: not performed

Clinically recognized: monomorphic VT (LBBB/inferior axis type)

# Patient 2

EPS: not performed

Clinically recognized: none

# Patient 3

EPS: monomorphic VT (RBBB type)

Clinically recognized: monomorphic VT (LBBB/superior axis type)

# Patient 4

EPS: monomorphic VT (LBBB/inferior and LBBB/superior axis type)

Clinically recognized: monomorphic VT (LBBB/superior axis type)

# Patient 5

EPS: monomorphic VT (LBBB/superior axis type)

Clinically recognized: monomorphic VT (LBBB/superior axis type)

**Supplementary Doc. 3**

**Findings of cardiac magnetic resonance and computer tomography among the ARVC patients with Brugada ECG pattern**

| Figure corresponding to the patient | CMR | | | |  | CT | |
| --- | --- | --- | --- | --- | --- | --- | --- |
|  | RV asynergy | RV LGE | fat tissue | LV LGE |  | RV asynergy | fat tissue |
| Patient 1 | Inferior, dyskinesis; anterior, severe. | none | none | none |  | diffuse severe | none |
| Patient 2 | Apex, dys-akinesis. | none | septum | none |  | - | none |
| Patient 3 | - | - | - | - |  | - | - |
| Patient 4 | Diffuse hypokinesis | none | atrial septum | none |  | none | septum |
| Patient 5 | Inferior and inflow, akinesis; diffuse hypokinesis | RVOT | Lateral, inferior | Latera, inferior |  | - | free wall |

CMR=cardiac magnetic resonance image, CT=computed tomography, RV=right ventricular, LGE=late gadolinium enhancement, LV=left ventricular
